# The impact of chemical pollution on the European eel (*Anguilla anguilla*) from a Mediterranean hypersaline coastal lagoon

**DOI:** 10.1007/s11356-023-27871-9

**Published:** 2023-06-08

**Authors:** Concepción Martínez-Gómez, Beatriz Fernández, Elena Barcala, Víctor García-Aparicio, Esther Jumilla, Ángel Gea-Pacheco, Víctor Manuel León

**Affiliations:** 1 Instituto Español de Oceanografía (IEO), CSIC, Centro Oceanográfico de Murcia, C/ Varadero 1, 30740 San Pedro del Pinatar, Murcia, Spain; 2 Centro de Edafología Y Biología Aplicada del Segura (CEBAS), CSIC, Campus Universitario de Espinardo. Espinardo 30100, Murcia, Spain; 3 Chemistry Faculty, University of Murcia, Campus Universitario de Espinardo, 30100 Murcia, Spain; 4 Sciences Faculty, University of Alicante, San Vicente del Raspeig Road. S/N, 03690 San Vicente del Raspeig, Alicante, Spain

**Keywords:** Biomarkers, Eel stocks, Mar Menor lagoon, Organic contaminants, Protected area

## Abstract

The European eel (*Anguilla anguilla)* is a critically endangered species. The impact of environmental contamination on this species has been highlighted as contributing to the decline in recruitment. The Mar Menor hypersaline coastal lagoon (SE Spain) is one of the most productive fisheries of European eel in Europe, making it a critical habitat for species conservation. The present study aimed to provide an initial overview of the impact of organic chemical contaminants on the European eel and the potential sublethal effects of chemical pollution on pre-migrating eels in this hypersaline habitat. We investigated muscle bioaccumulation of main persistent and hazardous organic contaminants (including some current-use pesticides) and genotoxicity, neurotoxicity, and xenobiotic detoxification system responses. The findings show that lagoon eels were exposed to high levels of legacy organochlorine contaminants, recently banned pesticides (chlorpyrifos), and some emerging chemicals. Some individuals surpassed the maximum levels of CBs authorized by the European Commission for human consumption. In this species, residuals of chlorpyrifos, pendimethalin, and chlorthal dimethyl have been reported for the first time. This field study provides relevant data to stock management and human health consumption and provides the first biomarker responses in European eel under permanent hypersaline conditions. Furthermore, the high frequency of micronuclei in peripheral erythrocytes of lagoon eels indicates sublethal genotoxic effects on the organism. Overall, the European eels growing and maturing in the Mar Menor lagoon are exposed to toxic and carcinogenic chemicals. The lack of seafood safety regulations for human consumption for some legacy chemicals that were measured in high concentrations in our study requires special action. Further biomonitoring and research are recommended to protect the animal, public, and environmental health.

**Supplementary Information:**

The online version contains supplementary material available at 10.1007/s11356-023-27871-9.

## Introduction

The European eel (*Anguilla anguilla*, Linnaeus 1758) is a diadromous fish that is critically endangered, according to the International Union for Conservation of Nature’s Red List (Pike et al. 2020). Currently, its recruitment is low, and its stock status remains critical (Aalto et al. 2016; ICES 2019). Mediterranean coastal lagoons represent one significant continental habitat where European eel grows as juveniles and mature adults (Cataudella et al. 2014). Such is the case for the Mar Menor lagoon (MML) (SE Spain), a Specially Protected Area of Mediterranean Importance since 2001 (SPAMI's list; RAC/SPA 2020), which is a singular habitat for European eels as it has permanent hypersaline waters (42–47 PSU). In addition, the MML is home to one of Europe’s greatest yellow and silver eel fisheries. Despite decreased catches in recent decades, the MML remains a critical continental habitat for conserving the European eel (Barcala et al. 2022). Unfortunately, human activities such as extensive agricultural and livestock operations, tourism, significant urban growth, and the relics of historical metal mining activities have all had a significant impact on environmental status during the previous 50 years, putting the MML in jeopardy (Marín-Guirao et al. 2005; Conesa and Jiménez-Cárceles 2007; Moreno-González et al. 2013, 2015; Moreno-González and León, 2017; Belando-Torrente et al. 2019; Ruiz et al. 2020).

Multiple factors have been proposed as causes for the diminishing global European eel population (reviewed by van Ginneken and Maes 2005; van Ginneken 2017). Contaminants and their associated effects on the reproductive capacity of eels (e.g., chemical contaminants maternally transferred into gonads and eggs) have been studied as one factor contributing to recruitment decline and non-fishery mortality (Belpaire et al. 2019; Freese et al. 2017, 2019). Juvenile and adult eels tend to accumulate greater amounts than other fish species of environmental chemical contaminants, particularly persistent bioaccumulative toxic (PBT) compounds. Furthermore, it has been widely reported in the literature that pre-migrating European eel inhabiting chemically impacted environments show a high contaminant accumulation in their bodies (Van der Oost et al. 1996b; Belpaire and Goemans 2007; Couderc et al. 2015). This is due to their physiological and ecological characteristics (i.e., their high lipid body content, long life cycle, semelparous reproductive strategy, and benthonic ecology) (Roche et al. 2000; Bordajandi et al. 2003; Steendam et al. 2020). The European eel arrives in continental habitats (glass eel stage), and they remain in them (from 6 up to 20 years) until they migrate back (silver eel stage) to their spawning grounds (which are likely about 5000–7500 km away), probably lasting between 3.5 and 6 months of continuous swimming and the onset of sexual maturation (Amilhat et al. 2016; Miller et al. 2019). During their growing stage in continental habitats, the European eel undergoes a physiological and morphological transformation developing from yellow eel to the migratory silver eel stage (Durif et al. 2005). The morphological changes associated with silvering include a change in body color, an increase in eye size, and an increase in body fat (to a maximum of 25–30% of their body mass). Silver eels are believed not to feed during their spawning migration, and meanwhile, fat reserves are consumed, and the toxic bioaccumulated contaminants are mobilized and translocated, becoming more bioavailable (Robinet and Feunteun 2002; Sühring et al. 2015). Consequently, silver eels are more likely to experience adverse effects on their health and reproductive performance (Geeraerts and Belpaire 2010; Sühring et al. 2015).

The observed effects in the European eel caused by PBT contaminants in experimental exposure studies vary according to the type and concentration of individual pollutants and their mixtures. They include genotoxic damages and disturbances of the immune, reproductive, nervous, and endocrine systems (reviewed by Geeraerts and Belpaire 2010; Guilherme et al. 2010). For example, acute concentrations of organochlorine and organophosphorus pesticides in the water caused eels to show restlessness, erratic swimming, convulsions, loss of balance, mucus secretion, and pale color (especially with chlorpyrifos) before death (Ferrando et al. 1991).

Induction of cytochrome P4501A (CYP1A-mediated phase I metabolism) provides evidence of fish biotransformation of numerous persistent contaminants with planar conformation (Whyte et al. 2000). The occurrence of erythrocyte nuclear abnormalities such as micronucleus provides information on chromosomal damage and can be used to investigate the impact in fish of environmental genotoxic contaminants (Bolognesi et al. 2006). On the other hand, organophosphorus pesticides, PCBs, and certain pesticides such as hexachlorobenzene (HCB) and the *p,p*-dichlorodiphenyldichloroethylene (*p,p*´-DDE) are known neurotoxicants (Miodovnik 2011), and acetylcholinesterase activity (AChE) is recognized as a biomarker of neurotoxicity in fish (Burgeot et al. 2012). However, few field studies have examined contaminant-related biomarker responses in the European eel, and most have been carried out during the past decades in brackish or freshwaters (Van der Oost et al. 1996a; Livingstone et al. 2000; Doyotte et al. 2001; Corsi et al. 2005; Guimaraes et al. 2009). During the last decade, many EU member states have collected data on the chemical quality of eels in their water bodies (ICES 2011). However, there have been no specific studies on eels inhabiting MML to investigate contaminant-related effects, and the only bioaccumulation data available is for heavy metals (Romero et al. 2020).

The present study aimed to investigate the bioaccumulation of organic chemical contaminants and contaminant-related biomarker responses in pre-migrating European eel (*Anguilla anguilla*) from a hypersaline Mediterranean coastal lagoon. We analyzed polycyclic aromatic hydrocarbons (PAHs), polychlorinated biphenyls (PCBs), organochlorine and organophosphorus insecticides, and current-use pesticides in muscle of pre-migratory MML eels. In addition, we analyzed three contaminant-related biomarkers recommended for marine fish (UNEP/MAP 2018; Vethaak et al. 2017), including micronuclei frequency (MN) in peripheral erythrocytes, AChE activities in brain and muscle tissues, and hepatic ethoxyresorufin-*O*-deethylase (EROD) activity, covering important toxicity mechanisms (genotoxicity, neurotoxicity, and induction of CYP1A-mediated phase I metabolism detoxification system). In addition to contributing to stock management and conservation, our findings on MML eels will help assess environmental quality and protect human health.

## Material and methods

### Study area and sampling procedure

**Fig. 1:**
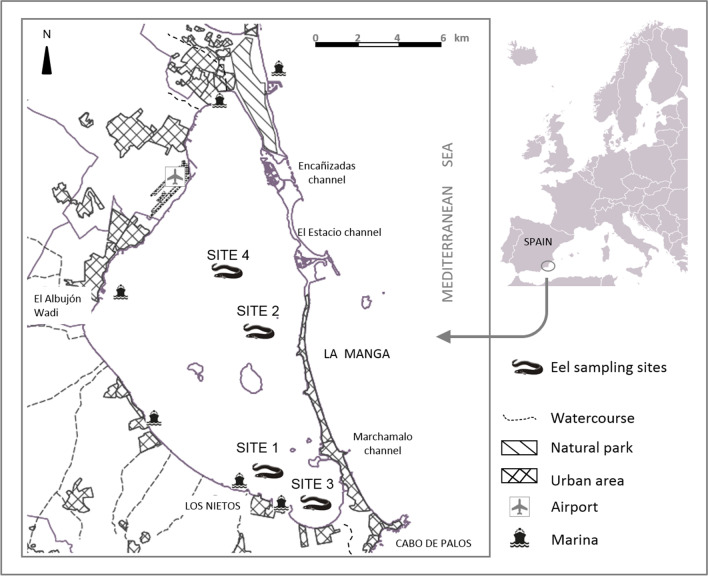
Location of European eel (*Anguilla anguilla*) sampling sites (S1, S2, S3, and S4) in the Mar Menor lagoon (Spain, South-west Mediterranean)

The Mar Menor is a coastal lagoon in Murcia (South-East Spain), adjacent to the Campo de Cartagena area, where intensive agricultural activity has taken place since the nineteen eighties. A sandbar of 22 km in length separates the lagoon from the Mediterranean Sea (Fig. 1). Water exchange and, therefore, the passage of species between the MML and the Mediterranean Sea occur through three channels and two navigable canals, one of them sheltering the largest marina in the lagoon (Estacio Canal). During the year, seawater temperature (SWT) usually ranges from 10 to 30 °C and salinity from 42 to 47 PSU. The total lagoon surface area is nearly 135 km^2^, and urban and recreational infrastructures mostly occupy the coastal area (length of 70 km). The lagoon has a maximum depth of 7 m (mean depth range between 3 and 4 m), and the water residence time in the lagoon is almost 1 year. The southern part of the MML receives metal mining waste through water runoff, and overall surface sediments have a lower organic matter (total organic carbon about 3.8%) than in the central area of the lagoon (6–8%). The central area is under the influence of the marine traffic from the Estacio Canal (east bank) and of the main collector watercourse of the drainage basin named “El Albujón” (west bank) (Fig. 1). There is a regular flux of groundwater feeding this watercourse that is only continuous in the last few kilometers, and it receives agricultural runoff, treated effluents, and brackish water effluents. As a result of these circumstances, MML acts a sedimentary trap of metal and organic chemical contaminants.

It is stated that most of the glass eel specimens reaching the MML spent their entire life dwelling in the hypersaline waters (42–44 PSU) before migrating for reproduction (Peñalver et al. 2015). The total number of fish used in the present study (*N* = 58) was obtained shortly after their capture from a local fishery aimed at being sold for human consumption over two fishing seasons. The fish were caught using fyke nets (Paranza) in spring and long lines in winter. Sampling sites were located in the southern sub-basin (S1 and S3) and the central area of the lagoon (S2 and S4). The timing and location of the fishing gear used in the MML were, therefore, factors that determined the sampling site and the sampling time for the eels in this study. The bottoms of all sampling sites were covered by *Cymodocea nodosa* and *Caulerpa prolifera* meadows (Fig. 1). Eels were collected in April 2014 from site S1 (SWT = 16.0 °C) and in January–February 2015 from sites S2, S3, and S4, (SWT ranged from 13.8 to 14.6 °C). In order to minimize the effects of environmental and biological confounding factors on biomarker responses, only eels collected in spring by fyke nets were used for biomarker analysis. After being captured, eels were transferred to the laboratory and held in 500-L flow-through holding tanks (using Mar Menor seawater) for a maximum of 2 days to stabilize their physiological conditions. Fish were handled following the principles and regulations for protecting animals used for scientific purposes (RD 53/2013), except for chemical anesthetics, which could interfere with some biomarkers analysis (Topic Popovic et al. 2012; Teles et al. 2019). Shortly before blood extraction, the physical method of gradual cooling was used to achieve a deep plane of anesthesia. This method has proven helpful during short-term procedures, such as intraperitoneal injections in fish (Wilson et al. 2009; Collymore et al. 2014). Individual blood samples were collected from the caudal vein and smeared on clean glass slides. Immediately after blood sampling, fish were killed by cervical severance. Bile fluid was collected by a disposable syringe after opening up the body cavity of the fish. Bile samples were immediately frozen in microtubes and stored (− 80 °C) until analysis. After that, the brain, muscle, liver, and gonads were dissected, weighed, and stored for subsequent biological analysis and the calculation of the physiological parameters. Liver and brain tissue for biomarker determinations were stored in liquid nitrogen at − 196 °C. Gonads were fixed in a formaldehyde-buffered solution for 24 h and subsequently stored in ethanol 70% until further processing. Finally, the otoliths were removed, cleaned, and dried, and the sagittal otoliths stored. Eviscerated eels were stored at − 20 °C until further processing for chemical analysis. Filet samples were always excised from the right side at the anus level. For each specimen, the total length (L_T_) (mm) and eviscerated body weight (W_E_) (g) were recorded. Additionally, the maximum pectoral fin length (L_PF_) and horizontal (D_h_) and vertical diameters (D_v_) of left eyes were measured (in mm) using a digital caliper (0.01–150 mm).

### General physiological indicators

After fixation, gonads were embedded in paraffin, and 5-μm-thick slices were cut and stained with hematoxylin–eosin following standard methods. Fish age, sex, and developmental stage were assessed by a histological examination of the otoliths and gonads following the procedures described in Barcala et al. (2022). In short, otoliths were cleared in alcohol 96% and microscopically examined (40 × magnification) with reflected light against a dark background (ICES 2009). Two readers established fish age by counting the winter rings on each left otolith. Fulton condition factor (*K*), hepatosomatic index (HSI), and gonadosomatic index (GSI) were calculated. Briefly, *K* was individually calculated as *K* = 100·W_E_ (g)/L_T_^3^ (cm). HSI and GSI were also individually calculated as HIS = 100·liver weight (g)/W_E_ (g) and GSI = 100·gonad weight (g)/ W_E_ (g). The ocular index (OI) and the fin index (FI) were calculated using the following formulas: OI = ((D_v_ + D_h_)/4)^2^·(π/L_T_)·100 (Pankhurst 1982) and FI = (L_PF_/L_T_)·100 (Durif et al. 2005). The GSI is related to the level of gonad maturation and OI and FI with the metamorphosis process development. Eels were considered silver eels when GSI > 0.6, OI ≥ 6.5, and FI ≥ 4.9, while they were considered silvering eels if only some of the characters were present and yellow eels if none of them were present. The lipid content of each muscle sample was determined individually using Soxhlet extract obtained for organic contaminant analyses. Briefly, 10 mL of the extract was transferred to a weighed evaporating dish, and the solvent was evaporated and expressed as mg lipid·g^−1^ muscle tissue wet weight (w.w.).

### Biomarker analysis

Biomarker analysis included acetylcholinesterase activity (AChE) in brain and muscle tissue, ethoxyresorufin-*O*-deethylase (EROD) activity in hepatic tissue, MN frequency in peripheral erythrocytes, and erythrocytic nuclear abnormalities (ENA). In addition, the analysis of EROD activity in liver microsomes was performed following the fluorometric method described by Burke and Mayer (1974) and adapted to a microplate reader (Martínez-Gómez et al. 2017). EROD activity is expressed as picomoles of resorufin generated per minute reaction time and milligrams of microsomal proteins (detailed information is provided in supplementary material). AChE activity was analyzed in the brain and muscle tissue following the method of Ellman et al. (1961), modified to a fluorometric method, and adapted to a microplate reader as described in Martínez-Gómez et al. (2017). AChE activity is expressed as nanomoles of thiocoline generated per minute reaction time and milligrams of cytosolic proteins (detailed information is provided in supplementary material). MN frequency was assessed following the method and identification criteria described in Bolognesi et al. (2006). Glass slides containing smeared blood fish samples were dried overnight, fixed with methanol for 30 min, and then stained with acridine orange. Only cells with intact cellular and nuclear membranes were considered. Additionally, erythrocytic nuclear abnormalities (ENAs) were also considered following the procedure described in Pacheco and Santos (2001). Nuclear lesions observed were scored into one of the following categories: micronuclei (M), lobed nuclei (L), dumbbell-shaped or segmented nuclei (S), and kidney-shaped nuclei (K). About 5000 erythrocytes per animal were analyzed by a fluorescence microscope (OLYMPUS BX43) under oil immersion at 1000 × magnification. Results of MN and ENA were expressed as ‰ (detailed information provided in the “Supplementary information”).

### Chemical analysis

PAHs and their metabolites, organochlorine compounds, organophosphorus, and other current-use pesticides were among the four environmental chemical pollutants studied. Fish were individually analyzed for each group of compounds. Approximately 2 g of lyophilized muscle tissue samples were Soxhlet extracted, and the final extracts were evaluated. The PAH analyses were conducted using high-performance liquid chromatography with fluorescence detection (HPLC) (Alliance Waters 2695; fluorescence detector Waters 2475), following the methodology described in León et al. (2014). The following fourteen hydrocarbons were analyzed: fluorene, phenanthrene, anthracene, fluoranthene, pyrene, benzo[a]anthracene, chrysene, benzo[e]pyrene, benzo[b]fluoranthene, benzo[k]fluoranthene, benzo[a]pyrene, benzo[g,h,i]perylene, dibenzo[a,h]anthracene, and indeno[1,2,3-c,d]pyrene (detailed information provided in supplementary material). Vertebrates metabolize PAHs rapidly, and the main environmental PAH metabolite detected in fish bile is 1-hydroxypyrene (1-OHPyr), contributing up to 76% of the sum of PAH metabolites (Kammann et al. 2013). Concentrations of pyrenol (1-hydroxypyrene) and phenanthrol (1-hydroxyphenanthrene) were quantified in eel bile samples performing HPLC analysis and following the method described by Kammann (2007), with minor modifications (detailed information provided in the “Supplementary information”).

Organochlorine and organophosphorus compounds, and current-use pesticides were analyzed by gas chromatography-mass spectrophotometry (GC–MS) using a GC 6890N coupled with an Inert XLD 5975 quadrupole mass spectrometer (Agilent) (detailed information provided in the “Supplementary information”). The analysis included the quantification in muscle tissue of the following compounds: polychlorinated biphenyls (PCBs) IUPAC No. 28, 52, 101, 105, 118, 138, 153, 156, and 180, organochlorine pesticides dichloro-diphenyl DDXs (*op′*-DDT, *pp′*-DDT, *pp′*-DDE, *pp′*-DDD), hexachlorocyclohexane isomers (α-HCH, β-HCH, and γ-HCH), HCB, cyclodiene insecticides (aldrin, dieldrin, endrin, and isodrin), trans-nonaclor (T-NNC), organophosphorus insecticides chlorpyrifos, fenchlorphos, trichloronate, and prothiofos and the herbicides chlorthal-dimethyl (DCPA, Dachtal), and pendimethalin. Detection limits (DLs) of the contaminants analyzed are provided in the “Supplementary information” (Table S1).

### Data analysis

Statistical analyses were carried out using the SPSS statistical package (SPSS v. 15.0). Data were log-transformed (Log X) whenever required. The normality of data was tested by using Shapiro–Wilk. Before parametric analysis, the homogeneity of variances was checked using Levene’s test. According to the data nature, differences were checked by using parametric (*t*-test of the mean, 1-way ANOVA tests) or non-parametric tests (U-Mann–Whitney or Kruskal–Wallis). If significant differences were found, Tukey, Tukey-b, or Tamhane T2 tests were applied for sampling site comparisons. A setting of α = 0.01 was used to compensate for the increased likelihood of type I error caused by the unbalanced sampling of eels (Kingsford 1998). Mean concentrations of contaminants were calculated only for groups where concentration data above the DL represented ≥ 50% of samples. In these groups, half the DL was used for censored data (< DL). According to the data nature, Pearson and Spearman Rho correlation coefficients were calculated whenever required and where data were available. Contaminant concentrations in silvering eels caught at different sites/areas of the MML were compared whenever possible. Contaminant concentrations and biomarker responses in eels at different life stages (yellow/silvering/silver) were also tested for differences. To date, the assessment criteria of contaminant-related biomarkers in *Anguilla anguilla* have not been established. Therefore, we compared our data with similar data from field studies conducted elsewhere on European eel. Observed chemical concentrations were assessed against established European thresholds for human consumption (Commission Regulation (EU) Nº 1259/2011), environmental assessment criteria (EAC in fish) where possible (OSPAR Commission 2021), and environmental quality standards (EQS in fish) (European Commission 2014). In the framework of international assessment and advice, the International Council for the Exploration of the Sea (ICES) has developed the Eel Quality Index for Contaminants (EQI) (ICES 2012). EQI is derived from the Eel Quality Classes proposed by Belpaire and Goemans (2008). In our study, the distribution of ∑PCB (CB 28, 52, 101, 118, 138, 153, and 180) and ∑DDTs (sum of *p,p´*-DDT, *p,p´*-DDE, and *p,p´*-DDD) quality classes were calculated using data of yellow and silvering eels from the Mar Menor (*N* = 18) and following the procedure and reference values established by Belpaire and Goemans (2008).

## Results

### Catch results, fish parameters, and indices

Silvering eels accounted for more than half of the catch (55%), followed by yellow eels (28%) and silver eels (17%). The capture size ranged from 9 to 19 individual eels per site (S1 = 19; S2 = 9; S3 = 15; S4 = 15). Yellow eels were primarily derived from S1 (southern sub-basin), and silver eels from S4 (central sub-area) of the lagoon (Table 1). Detailed information on the sampling sites and fish captures is provided in Table S2.

**Table 1:** Biological parameters (mean ± SE) of the European eels (*Anguilla anguilla*) sampled from Mar Menor lagoon (SE Spain)

Eel stages	*N*	Length (cm)	Eviscerated weight (g)	Age (years)	Extractable lipid (mg·g^−1^w.w.)	I_F_	I_O_	*K*	HSI	GSI
		Mean ± SE	Mean ± SE	Mean ± SE	Mean ± SE	Mean ± SE	Mean ± SE	Mean ± SE	Mean ± SE	Mean ± SE
Yellow eels	16	46.55^a^ ± 1.05	135.06^a^ ± 10.84	3.1^a^ ± 0.2	110.6^a^ ± 16.0	4.14^a^ ± 0.06	4.92^a^ ± 0.25	0.13^a^ ± 0.00	1.65^a^ ± 0.14	0.40^a^ ± 0.10
Silvering eels	32	62.65^b^ ± 0.34	387.95^b^ ± 26.15	4.6^b^ ± 0.3	223.3^b^ ± 13.01	4.58^b^ ± 0.08	8.45^b^ ± 0.37	0.15^b^ ± 0.00	1.36^a^ ± 0.09	0.73^a^ ± 0.10
Silver eels	10	69.39^c^ ± 1.64	562.50^c^ ± 39.68	4.9^b^ ± 0.5	256.9^b^ ± 20.31	5.22^c^ ± 0.07	11.70^c^ ± 0.76	0.17^c^ ± 0.00	1.20^a^ ± 0.07	1.58^b^ ± 0.08

*SE* = standard error of the mean; *w.w.* = wet weight); *I*_*F*_ = Fin Index; *I*_*O*_ = Ocular index; *K* = Fulton Condition Index; *HSI* = Hepatosomatic Index; GSI = Gonadosomatic Index. Lowercase superscripts indicate inclusion to subgroups (Kruskal–Wallis and Tamhane T2 post hoc tests; ANOVA 1-way and Tukey-b post hoc test; α = 0.01)

As expected, lipid content was significantly lower in yellow than in silvering and silver eels (ANOVA 1-way, *p* value = 0.000; Tukey-b post hoc test) (Table 1). In addition, condition factor *K* showed significant differences between the three life stages, lowest in yellow and highest in silver eels (ANOVA 1-way, *p* value = 0.000; Tukey-b post hoc test). Eels at different life stages displayed similar HIS values, indicating a similar state of energy reserves in all specimens analyzed (ANOVA 1-way, *p* value = 0.059), even though HSI decreased with the maturation stage.

### Biomarker responses

**Fig. 2:**
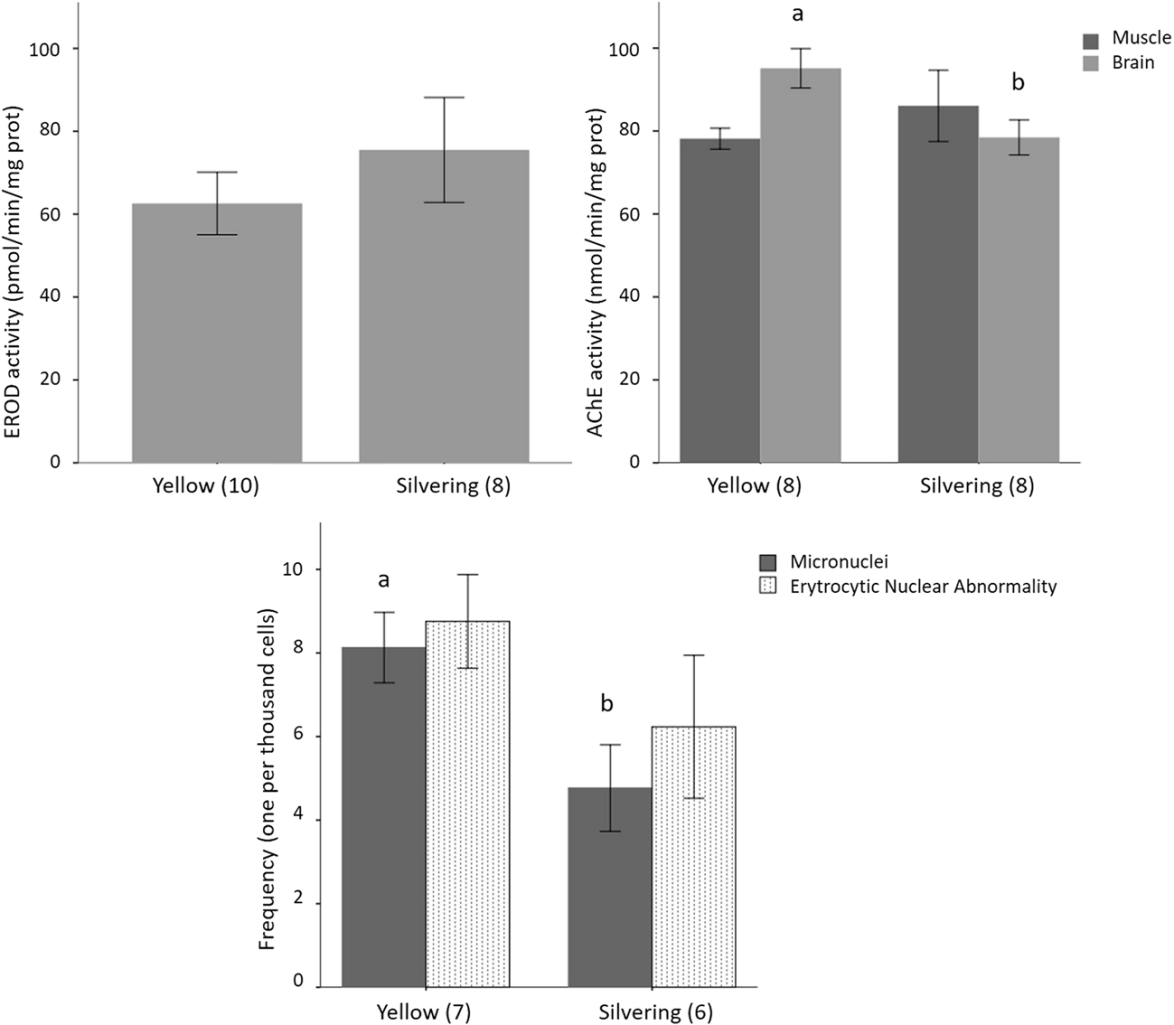
Biomarker responses (mean ± SE) in European eel (*Anguilla anguilla*) caught in spring from the Mar Menor lagoon (SE Spain) at yellow and silvering stages. Lowercase superscripts indicate inclusion in subgroups (*t*-test for the mean; *p* value < 0.05). Sampling size indicated between parentheses

Biomarker responses were assessed in yellow and silvering eels captured in April 2014 from the southern sub-area of the lagoon (Fig. 2). Except for two specimens (undefined sex), all were classified as females. Hepatic EROD activity varied from 53.6 to 83.0 pmol·min^−1^·mg^−^1 microsomal protein (confidence interval of the mean (95% CI)) and was similar between yellow (*N* = 10) and silvering eels (*N* = 8) (*t*-test for the mean; *p* value = 0.497). The other variables (age, length, weight, lipid content in muscle, *K*, GSI, and HSI) did not show any significant correlations with EROD activity (*N* = 18; *p* > 0.05). Overall, yellow (*N* = 8) and silvering eels (*N* = 8) had similar AChE activities in muscle tissue (72.7–91.6 nanomol·min^−1^·mg^−1^ protein; 95% CI); (*t*-test for the mean; *p* value = 0.402). However, AChE in the brain was higher in yellow (*N* = 8) than in silvering eels (*N* = 8) (*t*-test for the mean; *p* value = 0.020) (Fig. 2). Furthermore, brain AChE was inversely correlated with the age of the specimens (*N* = 16; Spearman-Rho coefficient =  − 0.687; *p* value = 0.002), the T_L_ and W_E_ of individuals, and *K* (Pearson’s coefficient >  − 0.57; *p* value < 0.05). The other variable lipid content in muscle, GSI and HSI, had no significant correlations with the brain AChE activities (*N* = 16; Pearson’s correlations; *p* > 0.05). The frequency of MN in eel erythrocytes ranged from 3 to 11‰ (mode of 3.2‰; *n* = 13), and it was significantly higher in yellow (*N* = 7) than in silvering eels (*N* = 6) (*t*-test for the mean; *p* value = 0.027). The predominant nuclear lesions after MN were segmented nucleus (S).

### Bioaccumulation of organic contaminants

#### PAHs and PAH metabolites

The bulk of PAH homologues was found in low concentrations in eel muscle (Table 2). Overall, the lipid content did not correlate with PAH concentrations in muscle tissue; the only exception was a weak correlation with indeno[1,2,3-c,d]-pyrene (Spearman-Rho coefficient = 0.397; *p* value = 0.008). Overall, PAH concentrations were similar in yellow (*N* = 7), silvering (*N* = 26), and silver (*N* = 10) eels from the lagoon (*p* values > 0.01; Kruskal–Wallis and Tanhane T2 post hoc tests; ANOVA 1-way and Tukey-b post hoc test; α = 0.01). The highest bioaccumulation of PAHs was mainly found for 3- and 4-ring PAHs (phenanthrene, fluorene, anthracene, fluoranthene, pyrene, and anthracene), except indeno[1,2,3-c,d]-pyrene, which was also found at high concentrations in some silvering and silver specimens (up to 22.2 µg·Kg^−1^ w.w.). Overall, 1-OHPyr values were significantly higher in eels sampled in spring (*N* = 8) (0.82 ± 0.07 ng·µL^−1^ bile) than in winter (*N* = 22) (0.31 ± 0.04 ng·µL^−1^ bile) (*t*-test for the mean; *p* value = 0.000). PAH metabolite concentrations in bile were not associated with EROD activity (*N* = 17), AChE activity (*N* = 15), and MN frequency (*N* = 12) in eels (*p* value > 0.3 in all cases). The silvering eels caught at different sites or sub-basins showed no clear spatial pattern in PAH concentrations and PAH metabolites (Table S3). In all analyzed samples, phenanthrol concentrations were below the detection limit (DL = 1.07 ng·µL^−1^). There were no significant correlations between the individual’s parental PAHs or 1-OHPyr metabolite concentrations and the eel’s weight, length, and age (*p* value > 0.05).

**Table 2:** Concentration (mean ± SE) and maximum value of polycyclic aromatic hydrocarbons (PAHs; µg·Kg^−1^ wet weight) in muscle and OH-PAH metabolites in bile (ng·µL^−1^ bile) of European eels (*Anguilla anguilla*) from Mar Menor lagoon (SE Spain)

	Yellow	Silvering	Silver	Total
PAHs	*N* = 7	*N* = 26	*N* = 10	*N* = 43
Fluorene	0.47 ± 0.19 (1.5)	0.7 ± 0.08 (1.46)	0.51 ± 0.11 (1.02)	0.62 ± 0.07 (1.5)
Phenanthrene	1.35 ± 0.49 (4.08)	2.04 ± 0.2 (4.13)	1.68 ± 0.14 (2.49)	1.84 ± 0.15 (4.13)
Anthracene	0.16 ± 0.09 (0.66)	0.32 ± 0.06 (0.97)	0.05 ± 0.03 (0.28)	0.23 ± 0.04 (0.97)
Fluoranthene	0.97 ± 0.68 (5)	0.73 ± 0.1 (2.15)	0.46 ± 0.09 (0.7)	0.71 ± 0.13 (5)
Pyrene	0.81 ± 0.65 (4.73)	0.9 ± 0.22 (4.5)	0.4 ± 0.16 (1.81)	0.77 ± 0.17 (4.73)
Benzo(a)anthracene	0.06 ± 0.02 (0.12)	0.06 ± 0.01 (0.21)	0.12 ± 0.02 (0.18)	0.07 ± 0.01 (0.21)
Chrysene	BDL	BDL	BDL	BDL
Benzo(e)pyrene	BDL (0.02)	0.15 ± 0.07 (1.14)	0.25 ± 0.12 (1.2)	0.15 ± 0.05 (1.2)
Benzo(b)fluoranthene	0.04 ± 0.01 (0.1)	0.05 ± 0.02 (0.42)	0.07 ± 0.02 (0.16)	0.05 ± 0.01 (0.42)
Benzo(k)fluoranthene	BDL	BDL (0.04)	BDL (0.04)	BDL (0.04)
Benzo(a)pyrene	BDL	BDL	BDL	BDL
Benzo(g,h,i)perylene	BDL	BDL	BDL	BDL (0.39)
Dibenzo(a,h)anthracene	BDL	BDL	BDL	BDL
Indeno[1,2,3-c,d]9pyrene	BDL	2.12 ± 1.09 (22.24)	2.55 ± 2 (19.78)	1.87 ± 0.8 (22.24)
OH-PAHs	*N* = 12	*N* = 30	*N* = 7	*N* = 49
1-OHPhen	BDL	BDL	BDL	BDL
1-OHPyr	0.65 ± 0.12 (1.21)	0.44 ± 0.06 (1.14)	0.4 ± 0.05 (0.6)	0.49 ± 0.05 (1.21)

*SE* = standard error of the mean

#### Organochlorine compounds

PCBs, drins, trans-nonachlor, HCB, *p,p*′-DDT, and their degradation intermediates (DDXs) were found in muscle tissue in eels from the MML, while concentrations of isodrin and hexachlorocyclohexane isomers (α-HCH, β-HCH, and γ-HCH) were below the detection limit in all analyzed samples (Table 3; Table S1). The predominant dichlorodiphenyl compound (DDXs) detected was *p,p′*-DDE. Negative and moderate correlations were found between the concentrations of PCBs, *p,p′*-DDE, *p,p′*-DDD, and aldrin of the individuals and their weight and length (*p* value < 0.05) (Table S4), which can be explained by growth dilution. ∑9CB concentration was inversely correlated with the age of the individuals (Spearman-Rho coefficient =  − 0.539; *p* value = 0.021). Spatial comparison analyses were only possible using data from silvering eels. Highest concentrations of Dieldrin, CB 138, CB180, *p,p′*-DDD, and, *p,p′*-DDE were found in eels caught from S1 in Spring (1-way ANOVA; *p* value < 0.01; Tukey-b test) (Table S5). For the rest of analyzed compounds, concentrations between eels from different sampling sites were found similar (Table S5). Overall, the highest mean concentrations of these compounds were found in yellow eels, but significant differences among eel stages were not statistically proven. The lipid content did not correlate with the various organochlorine concentrations analyzed in this study, except T-NNC (Pearson coefficient *r* = 0.627; *p* value = 0.003) (Table S6).

**Table 3:** Concentrations (mean ± SE and maximum value) of organochlorine compounds (ng·g^−1^ wet weight) and organophosphorus and other current-use pesticides (ng·g^−1^ wet weight) in muscle tissue of European eel (*Anguilla anguilla*) from Mar Menor lagoon (SE Spain)

	Yellow	Silvering	Silver	Total
Sampling size	*N* = 4	*N* = 14	*N* = 2	*N* = 20
α-HCH	BDL	BDL	BDL	BDL
β-HCH	BDL	BDL	BDL	BDL
γ-HCH	BDL	BDL	BDL	BDL
HCB	0.20 ± 0.09 (0.39)	0.31 ± 0.07 (0.87)	0.20 ± 0.03 (0.23)	0.28 ± 0.05 (0.87)
Aldrin	0.57 ± 0.16 (1)	0.59 ± 0.21 (2.34)	0.17 ± 0.12 (0.29)	0.55 ± 0.15 (2.34)
Isodrin	BDL	BDL	BDL	BDL
Dieldrin	1.39 ± 0.10 (1.63)	1.78 ± 0.33 (5.51)	0.74 ± 0.55 (1.28)	1.6 ± 0.25 (5.51)
Endrin	0.64 ± 0.11 (0.84)	0.76 ± 0.05 (0.96)	0.89 ± 0.04 (0.93)	0.75 ± 0.04 (0.96)
Trans-nonachlor	0.52 ± 0.03 (0.56)	0.66 ± 0.05 (1.17)	0.59 ± 0.01 (0.6)	0.62 ± 0.04 (1.17)
CB28	0.08 ± 0.05 (0.18)	0.09 ± 0.04 (0.42)	BDL	0.08 ± 0.03 (0.42)
CB52	0.54 ± 0.16 (0.98)	0.56 ± 0.21 (2.29)	0.15 ± 0.1 (0.24)	0.51 ± 0.15 (2.29)
CB101	5.13 ± 0.93 (7.74)	5.73 ± 2.24 (22.48)	0.82 ± 0.05 (0.86)	5.12 ± 1.59 (22.48)
CB105	0.79 ± 0.16 (1.25)	0.95 ± 0.26 (3.82)	0.49 ± 0.12 (0.6)	0.87 ± 0.18 (3.82)
CB118	5.87 ± 1.22 (9.32)	5.86 ± 2.01 (24.18)	0.93 ± 0.56 (1.49)	5.37 ± 1.45 (24.18)
CB138	69.93 ± 19.07 (123.91)	67.96 ± 25.22 (257.67)	15.11 ± 7.7 (22.81)	63.07 ± 18.17 (257.67)
CB153	93.28 ± 27.11 (170.43)	88.46 ± 33.64 (348.23)	7.40 ± 3.11 (10.5)	81.32 ± 24.44 (348.23)
CB 156	3.02 ± 1.04 (5.95)	2.95 ± 1.15 (11.75)	0.44 ± 0.01 (0.44)	2.71 ± 0.84 (11.75)
CB 180	57.96 ± 20.96 (118.69)	55.63 ± 21.38 (210.82)	9.32 ± 4.49 (13.8)	51.46 ± 15.6 (210.82)
∑ 9CBs	226.91 ± 68.02 (421.93)	218.43 ± 82.62 (840.22)	32.78 ± 9.13 (41.91)	201.56 ± 59.86 (840.22)
∑ 6CBs _(1)_	236.59 ± 70.41 (438.45)	228.19 ± 85.98 (879.58)	34.63 ± 8.46 (43.09)	210.51 ± 62.28 (879.58)
pp-DDD	7.8 ± 1.39 (10.31)	6.35 ± 1.76 (22.36)	3.22 ± 0.84 (4.06)	6.33 ± 1.27 (22.36)
pp-DDE	146.35 ± 29.36 (220.91)	150.08 ± 53.36 (724.36)	46.16 ± 19.8 (65.95)	138.94 ± 37.99 (724.36)
op-DDT	1.29 ± 0.31 (2.03)	1.91 ± 0.29 (4.82)	1.86 ± 0.03 (1.89)	1.78 ± 0.21 (4.82)
pp-DDT	0.61 ± 0.06 (0.71)	0.84 ± 0.2 (3.27)	0.59 ± 0.08 (0.66)	0.77 ± 0.14 (3.27)
∑ DDTs _(2)_	156.05 ± 30.03 (232.63)	159.18 ± 55.30 (750.51)	51.82 ± 20.53 (72.35)	147.81 ± 39.36 (750.51)
Chlorpyrifos	2.95 ± 0.54 (3.64)	6.22 ± 1.45 (20.18)	6.28 ± 3.74 (10.03)	5.57 ± 1.09 (20.18)
Pendimethalin	BDL	0.88 ± 0.16 (2.11)	1.47 ± 0.14 (1.61)	0.8 ± 0.14 (2.11)

^(1)^Sum of PCB28, PCB52, PCB101, PCB138, PCB153, and PCB180 according Commission regulation (EU) No 1259/2011 (maximum safe level ∑6CBs ≤ 300 ng·g^−1^ wet weight). *SE* = standard error of the mean; *BDL* = below detection limit

^(2)^Sum of *op*-DDT, *pp*-DDT, *pp*-DDE, and *pp*-DDD according Commission regulation (86/363/EEC—in meat) (maximum safe level ∑DDTs ≤ 1000 ng·g^−1^ wet weight)

#### Organophosphorus and other current-use pesticides

Chlorpyrifos (up to 20.18 ng·g^−1^ w.w.) was detected in all samples except one. This compound was the most bioaccumulated organophosphorus pesticide in eels, its concentrations unrelated to the age, weight, length, and lipid content of individuals (*p* value > 0.05) (Table 3). The pesticide pendimethalin was detected only in eels caught during winter. Chlorthal-dimethyl (Dacthal) was detected in four of the five spring samples and one of the 15 winter samples (Table 3). Fenchlorphos and prothiofos concentrations were below the DL in all samples analyzed. The concentration of chlorpyrifos was similar in silvering eels from different sites of the lagoon (*p* value > 0.05) (Table S5).

## Discussion

### Environmental contaminant exposure in eels from Mar Menor

In this study, the eels analyzed were predominantly at the silvering stage, meaning they underwent the metamorphosis process from the “yellow” to “silver” stage. Most of the analyzed eels were sexually undefined or female individuals, in agreement with previous studies indicating that females dominate the Mar Menor eel population (Barcala et al. 2022). The eel stage distribution obtained in our study agreed with the one obtained by commercial fisheries in the MML (Barcala et al. 2022). This metamorphosis (from yellow to silver) involves morphological and physiological changes (i.e., lipid accumulation) that prepare the fish for their trans-oceanic migration to the spawning grounds (Durif et al. 2005). An increased feeding activity has been described in European eels during the warmer months relative to weaker but still significant activity in winter (Tesch 2003; Costa-Dias and Lobón-Cerviá, 2008). Therefore, the higher concentration of certain chemicals (i.e., 1-OHPyr, 138CBs, 180 CBs, Endrin, *p,p′*-DDD, and *p,p′*-DDT) in eels caught in spring compared to winter in MML was likely due to seasonal differences of feeding activity. Concerning this subject, European eel (145 ± 22 g) collected near the end of spring from Vaccarés lagoon (France) were more contaminated than those caught in the winter, supporting our findings (Roche et al. 2002).

Most of the glass eel specimens reaching the Mar Menor stay and grow in the lagoon before migrating for reproduction (Peñalver et al. 2015). As expected, we found bioaccumulation of the major man-made PBT contaminants in pre-migrating eels, previously found in the sediment, water, and invertebrates from this lagoon (León et al. 2013, 2017; Moreno-González and León 2017). In addition, spatial analysis of bioaccumulation values suggests some mobility of the eels across the MML. A recent study showed non-migrant eel movements back and forth across the channel that connects the Bages-Sigean coastal lagoon (France) with the Mediterranean Sea (Lagarde et al. 2021). Even if that could also be the case for eels living in the MML, exposure to contaminants is clearly higher in the lagoon than outside it, given the characteristics of the sedimentary basin. Consequently, bioaccumulation of contaminants found in eels from the MML is a direct consequence of this ecosystem’s poor chemical environmental state.

Marine fish drink considerably more water than fish from freshwaters to regain the water lost by osmosis. Therefore, the exposure to waterborne contaminants and contaminated fish prey can be higher in eels inhabiting polluted hypersaline waters, such as MML, than in eels inhabiting polluted brackish or freshwater habitats. In this regard, we found that concentrations of some organochlorine compounds were one order of magnitude higher in eels from the MML than from other polluted freshwater habitats of Spain (Mean values ∑6PCBs = 26.6 ng·g ^−1^ w.w.; *p,p’*-DDE = 29.9 ng·g ^−1^ w.w) (Bordajandi et al. 2003). Nonetheless, organochlorine compound bioaccumulation found in eels from MML was quite similar to data obtained in other European field studies (Corsi et al. 2005; Ferrante et al. 2010; Malarvannan et al. 2014; Couderc et al. 2015). Concentrations of *p,p′-*DDE (up to 724.36 ng·g ^−1^ w.w.), and HCB (up to 0.87 ng·g ^−1^ w.w.) were likewise exceptional in several yellow and silvering MML specimens. Degradation intermediate *p,p′*-DDE is the most abundant DDX found in sediments from the MML (León et al. 2017). It is well known that DDE is transferred to benthic fish in higher concentrations through the sediment (Sakurai et al. 2009). Consequently, the high concentration of this contaminant in eels is explained by the burrowing behavior into the substrate during the daylight of this species and the *p,p′*-DDE contamination in MML sediments. Our findings also revealed that the youngest and smallest yellow and silvering eels from the MML were the most contaminated by organochlorine compounds (with particular reference to PCBs). Authors investigating eels from Vaccarés lagoon (France) had similar findings, supporting ours. These findings were linked to alterations in energy metabolism (an increase in energy demand and concomitant reduction of glycogen content) caused by exposure to organochlorine insecticides during an initial stress response, followed by an adaptive increase (recuperation phase) (Roche et al. 2000). This hypothesis is supported by the lowest values of fish condition (*K)* observed in yellow eels from the MML. On the other hand, our results revealed that current-use pesticides, with particular reference to chlorpyrifos (insecticide used to control pests in agriculture activities and to control mosquitoes for public health purposes and on golf courses), have reached the non-target organisms living in the MML (up to 20.18 ng·g^−1^ w.w. of chlorpyrifos in eel muscle tissue). This finding is concerning and consistent with previous results found in water and sediments from this lagoon (Moreno-González et al. 2013; Moreno-González and León et al. 2017). The bioaccumulation of current-use pesticides in eels seems to be linked with the seasonal (i.e., pendimethalin and dacthal) and year-round use (i.e., chlorpyrifos) of these compounds in the Mar Menor area. Our results also confirmed that eels from the MML are moderately exposed to PAHs. The biliary concentrations of 1-OHPyr in eels from the lagoon were similar to those found in yellow eels in moderately polluted European and Mediterranean habitats (Wariaghli et al. 2015). The lack of correlation between PAH concentration and lipid content in eel’s muscle tissue found in our study was previously described by Roche et al. (2002) in Vaccarès lagoon (France) eels, supporting our findings. That can be explained by a growth dilution effect and the detoxification pathways in fish.

**Fig. 3:**
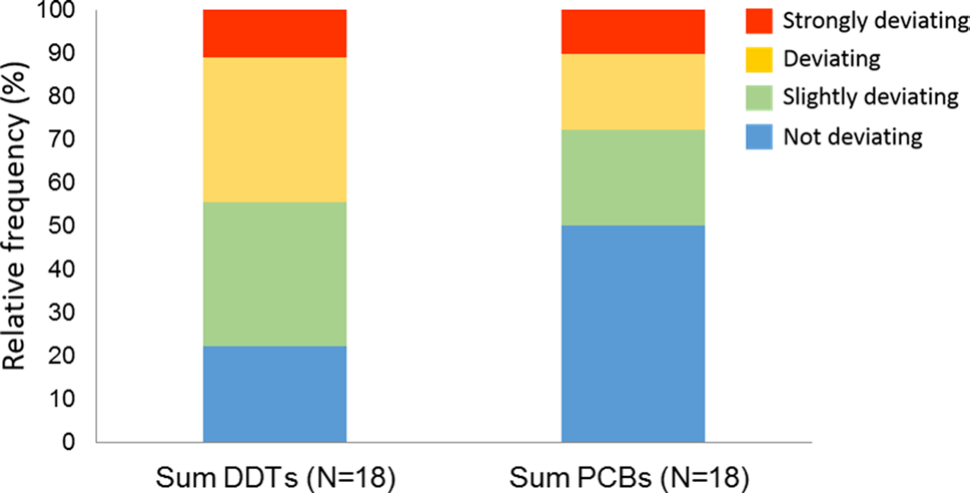
Distribution of ∑PCB and ∑DDTs quality classes in yellow and silvering eels from the Mar Menor Lagoon (*N* = 18; samples obtained from 4 sites), according to reference values established by Belpaire and Goemans (2008). Sum PCBs equals the sum of the 7 indicator congeners (CB 28, 52, 101, 118, 138, 153, and 180). Sum DDTs equals the sum of *pp*-DDT, *pp*-DDE, and *pp*-DDD

∑DDTs and ∑PCB levels were respectively deviating or strongly deviating from the reference values at respectively 44.4% and 27.8% of the samples (Belpaire and Goemans 2008) (Fig. 3). In fact, several individuals significantly surpassed the current maximum levels authorized for human consumption of CBs (Sum of CB28, CB52, CB101, CB138, CB153 and, CB180 > 300 ng·g^−1^ w.w.) according to Commission Regulation (EU) Nº 1259/2011 and overpassed the threshold values of Environmental Assessment Criteria of CB101, CB118, CB138, CB153, and CB180 (OSPAR Commission 2021) (Table S5). While the regulatory toxicity limits for single contaminants in seafood are intended to protect human health, it is widely recognized that the effects of the chemicals are often additive and sometimes synergistic. MML eel is sold in other parts of Spain and Europe, with the consumption of this species by the local population being negligible. According to European legislation (Commission Regulation (EU) No 1259/2011), the maximum safe level for the sum of PCB28, PCB52, PCB101, PCB138, PCB153, and PCB180 in the muscle meat of wild-caught eel (*Anguilla anguilla*) is 300 ng·g^−1^ wet weight. In our study, 20% of the eels analyzed exceeded this threshold. Previous research has shown that 13% of examined eels from MML contain levels of Pb in muscle tissue that are unsafe for human consumption (Romero et al. 2020). Concerns about human health have already been raised for chlorpyrifos (highest value found in MML eels of 20.18 ng·g^−1^ w.w.), notably concerning potential genotoxicity, developmental neurotoxicity, and detrimental effects on children’s health (EFSA 2019). The use of this insecticide was banned in Europe in 2020, and the European Member States endorsed a Commission proposal to reduce the Maximum Residue Levels (MRLs) for chlorpyrifos and chlorpyrifos-methyl in vegetables and fruits to the lowest level that can be determined by analytical laboratories (10 ng·g^−1^). Although Regulation (EC) No 1881/2006 and its amendments do not establish regulatory standards for *p,p′*-DDE, and HCB, concentrations of these contaminants were found one order of magnitude higher in this study than the background levels established in fish through regional cooperation (Robinson et al. 2017). The lack of seafood safety regulations for human consumption for some legacy chemicals (i.e., *p-p'DDE*, chlorpyrifos) that were measured in high concentrations in our study requires special action.

### Sub-lethal effects of chemical pollution in eels from Mar Menor

Overall, the highest concentrations of most contaminants analyzed in this study were found in eels sampled in Spring (S1). Therefore, contaminant-related biomarkers evaluated in eels from S1 integrate well the environmental exposure of this species to contaminants bioavailable in the lagoon and provide warning signals of the potential biological impacts these organisms may be experiencing. The use of biomarkers in European eel for environmental monitoring began and had its peak in the 1990s and 2000s, but at present, the number of published studies is still limited. The environmental assessment criteria for contaminant-related biomarkers in *Anguilla anguilla* have not yet been established. Because European eel does not undergo annual sex maturation, fluctuations in biomarker responses from non-pollutant factors are due to seasonal changes in seawater temperature and dietary habits rather than reproductive conditions. Our small data set did not allow proper correlation assessments between contaminant concentrations and biomarker responses. However, such correlations have been well described in several field and laboratory studies with European eel exposed to single and combined contaminants (van der Oost et al. 1997; Bonacci et al. 2003; Mariottini et al. 2003; Corsi et al. 2005).

Hepatic EROD activity is measured as a proxy of induction of the phase I metabolism for xenobiotics in fish. The highest levels of EROD activity (S9 liver fraction) in European eel have been found in summer and the lowest in winter, indicating that this system is temperature-dependent induced (Rotchell et al. 1999; Gorbi et al. 2005). Most field-based biomarker studies in pre-migrating eels have been performed in freshwater and brackish water continental habitats, and environmental and biological data in these studies are not always provided, limiting result comparisons. Nonetheless, mean EROD activities in yellow (65.5 ± 7.5 pmol·min^−1^·mg prot^−1^) and silvering eels (75.5 ± 12.7 pmol·min^−1^·mg prot^−1^) from the MML (Spring, 16 °C SWT) were higher than in eels from the Orbetello coastal lagoon (Italy), which is a brackish water impacted ecosystem that may reach hypersaline conditions in some areas, and higher than in eels from Tamar estuary (UK), which is considered as minimally polluted (Table 4). On the other hand, EROD activities in eels from the Thames estuary (UK), the Netherlands, and NW Portuguese estuaries were higher than in eels from MML, suggesting a moderate induction of EROD baseline levels in pre-migrating eels from the MML (Table 4). Supporting this suggestion, EROD values ranging from 25 to 40 pmol·min^−1^·mg^−1^ prot. have been reported in European eel from relatively unpolluted environments or control treatments (Table 4). Other authors have reported EROD microsomal activity ranging from 1 to 4 pmol·min^−1^·mg^−1^ prot. in small yellow eels (< 50 g) used as a control treatment under freshwater laboratory conditions and field-caging experiments (Table 4). However, in such contaminant-exposure experiments, the inhibitory effect of the contaminant carrier on EROD activity could partially explain the low EROD activity in control treatments, as described by Bonacci et al. (2003). Age-related factors (such as lifetime exposure/accumulation, food choice, and reproductive stage) may also have contributed to the observed variation. EROD activity was not strongly induced in MML eels (Table 4), but the effects of hormesis on EROD activity in eels inhabiting this lagoon should not be excluded.

**Table 4:** Hepatic microsomal EROD activity in the European eel (*Anguilla anguilla*) from field and laboratory studies (control treatments)

Area	Sampling season	Temperature ºC	Salinity PSU	Eel stage (n)	Total length (cm)	Wet weight (g)	EROD activity pmol/min/mg prot	Reference
Mar Menor lagoon (Spain)	Spring (April)	16	42–43	yellow (10)	42.5–48.0	98–148	45.5–79.6	This study
Mar Menor lagoon (Spain)	Spring (April)	16	42–43	silvering (8)	56.4–61.8	249.0–357.8	45.5–105.5	This study
Atlantic estuaries (Portugal)	Spring	18.7	6.1	yellow (20–40)	31.7–35.4	53.1–84.1	125–300	Guimaraes et al. 2009
Thames estuary (UK)	Spring (May)	n.p	0.1–1.2	n.p. (1–9)	32–44	114 ± 15	100 to 300	Doyotte et al. 2001
Tamar estuary (UK)	Summer (August)	n.p	Brackish and freshwater	n.p. (8)	34–43		37 ± 8*	Doyotte et al. 2001
Thames estuary (UK)	Spring (May)	n.p	8–17	n.p. (8–10)		114 ± 15	111 ± 24 to 355 ± 42	Livingstones et al. 2000
Amsterdam (Netherlands)	Summer (July)	20	Freshwaters	n.p. (≥ 10)	46–62	130–410	< 100 up to 1600	Van der Oost et al. 1996a, b
Orbetello Lagoon (Italy)	Summer (June)	n.p	From brackish to hypersaline waters	n.p. (15)	n.p	35	17.3 to 32.5	Corsi et al. 2005
Field caging (14 days)	Aude freshwater stream (France) April	12–15	Freshwater	yellow (6)	n.p	25	50	Fenet et al. 1998
Field caging (8 and 48 h)	Aveiro Harbour waters (Portugal) October	21	Saltwater	n.p. (5)	n.p	50	1.85	Maria et al. 2003
Laboratory exposure	Control treatment	15	35	Yellow (10)	46.6 ± 9.8	n.p	192 ± 14	Lemaire-Gony and Lemaire 1992
		17.9	Freshwater	yellow (6)	n.p	25	24.7 ± 5	Fenet et al. 1998
		17.5	Brackish waters	yellow (5)	n.p	15–55	15–50	Hewitt et al. 1998
		20–25	Freshwater	n.p. (5)	32.5—35	50.70	32.1 ± 8.3	Bonacci et al. 2003
		n.p	Freshwater	n.p. (20)	n.p	n.p	209 ± 85	Agradi et al. 2000
		20 ± 1	Freshwater	n.p. (5)	32.9 ± 2.6	72.6 ± 21.4	4.1 ± 2.5	Mariottini et al. 2003
		20	Freshwater	Yellow (5)	25.0 ± 3.0	30–50	< 1 to 8	Pacheco and Santos 2001; Teles et al. 2003

*n.p.* = info no provided. Asterisk indicates relatively unpolluted environment

AChE activities in eel from MML were rather similar in the brain (86.8 ± 3.7 nmol·min^−1^ mg prot^−1^) and muscle (82.1 ± 4.4 nmol·min^−1^ mg prot^−1^), which is consistent with previous research (Valbonesi et al. 2011). On the other hand, the analysis of individual data pointed out that brain AChE baseline levels could be related to eel growth and sexual maturation, with higher activities in yellow than in silvering and silver stages. This pattern has been observed in other fish species, such as *Callionymus lyra*, *Serranus cabrilla*, and *Mullus barbatus* (Galgani et al. 1992). Like EROD activity, seasonal differences in AChE activity have been described, with the highest activities occurring during the summer (Guimaraes et al. 2009; Burgeot et al. 2012). AChE in yellow eels from MML had almost three times higher brain activity than yellow eels sampled under similar environmental conditions (Spring; SWT = 18.7ºC) from several Portuguese estuaries (Guimaraes et al. 2009). Other authors have also found lower AChE activity than in our study, both brain, and muscle tissue, in yellow eels used as a control treatment (freshwater temperature 22 °C) (Fernández-Vega et al. 2002). Because of these findings, brain AChE activity in eels from MML did not appear to be inhibited by neurotoxicants, though more field data and experimental control data are needed to strengthen this assumption. The finding that eels from MML with higher concentrations of chlorpyrifos have reduced AChE activities was based on only 5 individuals and chlorpyrifos concentrations ranging from 3 to 7 ng·g^−1^ w.w. Despite this, further investigation is warranted since chlorpyrifos levels reached 20 ng·g^−1^ (w.w.) in eel muscle tissue in both spring and winter samples.

Yellow (8.1 ± 0.8‰) and silvering eels (4.8 ± 2.1‰) from MML had a higher MN frequency in peripheral erythrocytes than yellow eels collected in a relatively unpolluted area of Aveiro lagoon (Portugal) and eels used as control treatment in laboratory experiments (from 0.0 to 0.6‰) (Guilherme et al. 2010). Erythrocytic nuclear abnormalities (ENAs) obtained in MML eels were comparable to, in many cases, lower than ENA mean values reported in yellow eels employed as controls in field studies and laboratory exposure tests (Oliveira et al. 2008; Guilherme et al. 2010). However, because the frequency of each nuclear abnormality category is not included in most published studies, data cannot be directly compared. Eels exposed to glyphosate-based herbicides (Guilherme et al. 2010) had higher total ENAs than eels from MML, although showing different patterns, with most of the prevalent ENA linked to cytotoxicity events (kidney-shaped and lobed-shape nuclei) instead of genotoxicity events (MN and segmented nuclei). Since MN formation is a short-term response, the high MN frequency found in MML eels suggests they are subjected to exposure to genotoxic (potentially mutagenic) compounds. Cancer-prone benthic fish have the accumulation of contaminants associated with cancer prevalence (reviewed by Baines et al. 2021). Ribeiro et al. (2005) reported a range of liver and spleen lesions in European eel from the Camargue Reserve in southern France, including accumulation of melanomacrophage centers (MMC), necrotic areas, hepatic lipidosis, and various stages of tumor development (neoplasia). These lesions in eels and other bottom-dwelling fish species are associated with chronic exposure to toxic and carcinogenic chemicals (Vethaak et al. 1996; Baines et al. 2021). To determine if such toxicopathic lesions also occur in MML eels, histopathological analysis of their livers and spleens would be necessary.

### Need for further pollution biomonitoring with eels from Mar Menor

Unlike most species, the European eel only spawns once at the end of its life, and the pre-migrating eel’s health and fitness are thus crucial for successful reproduction. Our findings offer a snapshot of the chemical environmental state in which pre-migrating eels find themselves during their sedentary phase in the MML, which lasted roughly from 2008 to 2015 (age range of the studied eels). Unfortunately, the MML has been affected since 2016 by periodic algal blooms and severe dystrophic crises, which caused organisms die-offs, including European eel (the most dramatic events recorded in October 2019; personal communication) and a drastic reduction in biodiversity (Belando-Torrente et al. 2019; Caballero et al. 2022). Biomonitoring of MML eels, including histopathological examination, is therefore recommended.

## Conclusions

We found that pre-migrating eels inhabiting the Mar Menor lagoon bioaccumulate persistent and toxic contaminants in their tissues and show early warning signals of genotoxic effects. In accordance with the EU Marine Strategy Framework Directive, contamination levels found MML eels are relevant to human health consumption and environmental quality assessment, stock protection, and sustainable use of this species in coastal lagoons that flow into the Mediterranean Sea (European Eel Regulation EC No 1100/2007). Yellow and silvering eels from the MML were deviating from the reference values of ∑DDTs and ∑PCB established for this species. The high concentrations of PCBs and *p-p'DDE* found in eels from MML are consistent with those reported in eels from other polluted areas of Europe and exceeded the current maximum levels of PCBs authorized for human consumption in 20% of cases. The occurrence of several current-use pesticides in eels, such as pendimethalin and chlorthal dimethyl, and the recently banned chlorpyrifos, has been confirmed for the first time in this species.

This field study provides the first contaminant-related biomarker responses in pre-migrating European eel that grow and mature in a permanent hypersaline habitat. Biomarker results contribute to the knowledge of the natural limits of the variability of EROD and AChE activities and genotoxic responses in this specie that usually inhabits chemically contaminated habitats.

Additionally, the high frequency of micronuclei observed in blood erythrocytes suggests genotoxic and progenotoxic compounds are bioavailable to MML eels. The observed genotoxic effects in MML eels indicate health risks (e.g., carcinogenic and reproductive impairments) for this vulnerable species. Given its physiological and ecological characteristics, the European eel may be the best of all available fish species for monitoring chemical pollution in the MML for the WFD and MSFD. To adequately assess the potential health risks associated with the presence of regulated and non-regulated chemical contaminants, further biomonitoring studies and research are urgently needed.

## Supplementary Information

Below is the link to the electronic supplementary material.

Supplementary file1 (DOCX 63 KB)

## Data Availability

Data is available from the corresponding author upon request.
