# Supplementary material for: The impact of chemical pollution on the European eel (Anguilla anguilla) from a Mediterranean hypersaline coastal lagoon
Source: Environ Sci Pollut Res Int. 2023 Jun 8;30(33):80106–22. doi: 10.1007/s11356-023-27871-9 (PMC10344999; doi:10.1007/s11356-023-27871-9)
Supplement: Supplementary file 1 — Supplementary file1 (DOCX 63 KB) [file 11356_2023_27871_MOESM1_ESM.docx]

# Supplementary Material

**The impact of chemical pollution on the European eel (*Anguilla anguilla*) from a Mediterranean hypersaline coastal lagoon**

Martínez-Gómez C.^a*^, Fernández B.^a^, Barcala E.^a^, García-Aparicio V.^b^, , Jumilla E.^c^, Gea Pacheco A.^d^ and León V.M.^a^

*^a^ Instituto Español de Oceanografía (IEO), Centro Oceanográfico de Murcia (COMU-IEO), CSIC, C/ Varadero 1, 30740, San Pedro del Pinatar, Murcia, Spain*

*^b^ Centro de Edafología y Biología Aplicada del Segura (CEBAS-CSIC), Campus Universitario de Espinardo. Espinardo 30100, Murcia, Spain.*

*^c^ University of Murcia, Chemistry Faculty, Campus Universitario de Espinardo, 30100, Murcia, Spain.*

*^d^ University of Alicante, Sicences Faculty, San Vicente del Raspeig Road. s/n, 03690 San Vicente del Raspeig, Alicante, Spain*

* Contact:

[concepcion.martinez@ieo.csic.es](mailto:concepcion.martinez@ieo.csic.es)

Number of pages: 10; Number of supplementary tables: 7; Number of supplementary figures: 1

Abbreviations used in tables

- TOC: Total organic carbon
- FF: Fraction fine (<63µm)
- α-HCH: α-hexachlorocyclohexane
- HCB: hexachlorobencene
- TNNC: trans-nonaclor
- POPs: Persistent organohalogenated pollutants
- Σ4PAHs comprises the sum of phenanthrene, anthracene, fluoranthene and pyrene.
- Σ14PAHs comprises the sum of phenanthrene, anthracene, fluoranthene, pyrene, benzo[a]anthracene, chrysene, benzo[e]pyrene, benzo [b]fluoranthene, benzo[k]fluoranthene, benzo[a]pyrene, benzo[g,h,i]perylene, dibenzo[a,h]anthracene and indeno[1,2,3-c,d]pyrene.
- Σ9 CBs represent the sum of chlorinated biphenyl congeners No. 28, 52, 101, 105, 118, 138, 153, 156, and 180.
- Σ DDTs represent the sum of *p,p´*-DDE, *p,p´*-DDD and *p,p´*-DDT.

1. **Material and Methods**
   1. *Chemical analysis of PAHs*

Approximately two grams of lyophilized muscle tissue samples were Soxhlet extracted (10 h) with a hexane:acetone mixture (3:1). A clean-up of the sample extract was carried out with 10% partially deactivated alumina column, using hexane as eluent. The eluted fraction was concentrated in a rotary evaporator and re-dissolved to acetonitrile and then finally concentrated under a nitrogen stream to 1 mL. 20 µL of this extract were analysed by high performance liquid chromatography with fluorescence detection (HPLC). An HPLC Alliance Waters 2695 equipped with a fluorescence detector (Waters 2475) was used for the analysis, using specific excitation and emission wavelengths for each compound. The stationary phase was Waters PAH C18 5 mm 4.6 _ 250 mm column, whilst the mobile phase was an MeOH/H2O mix with a non-linear gradient regime (gradual increase of elution capacity of mobile phase). The following fourteen hydrocarbons were analysed: fluorene, phenanthrene, anthracene, fluoranthene, pyrene, benzo[a]anthracene, chrysene, benzo[e]pyrene, benzo[b]fluoranthene, benzo[k]fluoranthene, benzo[a]pyrene, benzo[g,h,i]perylene, dibenzo[a,h]anthracene and indeno[1,2,3-c,d]pyrene. The accuracy of the chemical and analytical procedures was internally assured by testing control, blanks and duplicated samples in each analytical series. The external accuracy of the analytical procedures was tested and controlled using certified material (freeze-dried marine sediment SRM-1941b and mussel tissue SRM-2977, National Institute of Standards & Technology, Department of Commerce, USA) and through satisfactory participation in the Quality Assurance of Information for Marine Environmental

- 1. *Chemical analysis of organochlorinated compounds*

About two grams of lyophilized samples were Soxhlet extracted (8 h) with a mixture (1:1) of n-pentane: dichloromethane. The clean-up of the sample extract was carried out using column chromatography on deactivated alumina (6% water), n-pentane being the eluent. The extracts were separated into two fractions using column chromatography on deactivated silica (3% water). Both organochlorinated extracts were finally analysed by GC–MS using a GC 6890N coupled with an Inert XLD 5975 quadrupole mass spectrometer (Agilent) and equipped with a PTV injector (CIS-4, Gerstel). TRB-5 (Tecknochroma, Spain) 5% diphenyldimethyl siloxane capillary (60m x 0.25 mm i.d. x 0.25 μm film thickness) was used. PCB 155 was added as an internal standard prior to GC analysis. The following compounds were considered for quantification: polychlorinated biphenyls IUPAC No. 28, 52, 101, 105, 118, 138, 153, 156, and 180, the organochloride pesticides dichloro-diphenyl-trichloroethane (DDTs), α-hexachlorocyclohexane (α-HCH), lindane (γ-HCH), hexachlorobenzene (HCB), cyclodiene insecticides (aldrin, dieldrin , endrin , and isodrin) and trans-nonaclor.

- 1. *Chemical analysis of Hydroxylated-PAHs metabolites in bile*

10 µL (individual or pooled sample) of fish bile was mixed with 230 µL of water (HPLC grade) and 10 µL of β-glucuronidase/arylsulfatase solution (30–60 U/mL). The mixture was subsequently incubated for 2 h at 37 °C on a heated shaker for enzymatic deconjugation. The reaction was stopped by the addition of 250 µL of cold methanol. After centrifugation (during ten minutes; 10000 rpm; 4ºC), the supernatants were subjected to HPLC analysis immediately. For quantification, 2-methil chrysene was used as internal standard (Dr.Ehrenstorfer, Augsburg). β-glucuronidase/arylsulfatase was bought from Roche (from *H. Pomatia*; 100,000 U/mL). The excitation/emission wavelength pairs for 1-OHPyr and 1-OHPhen were 346/384 and 256/380 nm, respectively (Alliance Waters 2695; fluorescence detector Waters 2475)

- 1. *Analysis of hepatic EROD activity*

Microsomes were prepared according to Förlin and Andersson (1985). All steps in microsome preparations were performed at 4 ºC. In brief, the livers were immersed in ice-cooled KCl buffer and then homogenized in 0.1 M phosphate buffer, pH 7.4, containing 1 mM dithiothreitol, 1 mM EDTA and 150 mM KCl, 25% w/v. Homogenates were centrifuged at 10,000g for 20 min and the resulting supernatant was centrifuged at 100,000g for 60 min. The supernatant, containing cytosol, was stored at -80ºC. The microsomal pellet was resuspended (1:1 liver w/v) in Tris–HCl buffer pH 7.4, containing 1 mM DTT, 0.1 mM EDTA and 20% glycerol. Resuspended microsomes were stored at -80ºC for subsequent assays. EROD activity was assayed in microsomes at an incubation temperature of 25 ºC according to the method of Eggens and Galgani (1992), a modified version of the fluorimetric method described by Burke and Mayer (1974), adapted to a microplate reader. Final concentrations in the well (350 µL) were 100 mM phosphate buffer pH 7.4, 2 µM 7-ethoxyresorufin and 0.25 mM NADPH. The progressive increase in fluorescence was monitored (excitation wavelength 535, emission wavelength 585; spectrofluorometer TECAN Spectrafluor Plus). EROD activities were normalized to either microsomal protein content determined following Lowry et al. (1951) and using bovine serum albumin as standard. The accuracy of the biochemical procedures was internally assured by testing control, blanks and triplicated samples in each analytical series. The external accuracy of the analytical procedures was tested and controlled using own reference material (marine fish liver homogenate from *Mullus* barbatus). This biological material was obtained from individuals caught from clean and polluted areas along the Spanish Mediterranean coast in 2014 and 2015 for same biomarker biomonitoring purposes (Martínez-Gómez et al, 2012, 2017).

- 1. *Analysis of Acetylcholinesterase activity (AChE) activity*

AChE activity was measured following the method of Ellman et al. (1961), modified to a fluorometric method and adapted to a microplate reader according to Bocquené and Galgani (1998). Tissue samples were homogenised (1:5 w/v fish brain; in a buffer solution (pH 7.0) containing 0.02 M NaH_2_PO_4_·H_2_O and 0.1% Triton X-100. Homogenates were centrifuged at 10,000 g for 20 min at 0-4 ºC and the supernatant subcellular fraction flash-frozen in liquid nitrogen before being stored at -80 ºC for subsequent AChE analysis. The enzymatic measurements were conducted at an incubation temperature of 25 ºC using the spectrofluorometer TECAN Spectrafluor Plus. AChE activities were normalized to cytosolic protein content determined following Lowry et al. (1951) and using bovine serum albumin as standard. The accuracy of the biochemical procedures was internally assured by testing control, blanks and triplicated samples in each analytical series. The external accuracy of the analytical procedures was tested and controlled using own reference material (marine fish brain and muscle tissue homogenates from *Mullus barbatus*). This biological material was obtained from individuals caught in clean and polluted areas along the Spanish Mediterranean coast in 2014 and 2015 for same biomarker biomonitoring purposes (Martínez-Gómez et al, 2012, 2017).

- 1. *Analysis of Micronuclei: identification criteria*

Micronuclei was defined as small round or oval intracytoplasmic bodies with a diameter 1/5–1/20 of the main nucleus and on the same optical plane as the major nucleus. The MN was not linked or connected to the main nucleus. Cells with more than four MN were discarded to exclude apoptotic phenomena. Nuclear anomalies, chromatin buds, lobes, invaginations, vacuoles, were recorded separately (Bolognesi et al., 2006). The external accuracy of the analytical procedures was tested and controlled using own reference material (marine fish blood slide samples from *Mullus barbatus*). This biological material was obtained from individuals caught in clean and polluted areas along the Spanish Mediterranean coast in 2014 and 2015 for same biomarker biomonitoring purposes (Martínez-Gómez et al, 2012, 2017). Erytrocytic nuclear abnormalities (ENA) was calculated as a mean value of the sum (M + L + S +K) for all the individual lesions observed. About 5000 erythrocytes per animal were analyzed by a fluorescence microscope (OLYMPUS BX43) under oil immersion at 1000× magnification.

**References:**

Bocquené, G. & Galgani, F., 1998. Biological effects of contaminants: cholinesterases inhibition by organophosphate and carbamate compounds. ICES Tech. Mar.Environ. Sci. 22, 1e13. https://doi.org/10.17895/ices.pub.5048

Bolognesi, C., Perrone, E., Roggieri, P., Pampanin, D.M. & Sciutto, A. (2006). Assessment of micronuclei induction in peripheral erythrocytes of fish exposed to xenobiotics under controlled conditions. Aquatic toxicology, 78, S93-S98. https://doi.org/10.1016/j.aquatox.2006.02.015

Burke, M.D. & Mayer, R.T. (1974). Ethoxyresorufin: direct fluorimetric assay of a microsomal O-dealkylation which is preferentially inducible by 3- methylcholanthrene. Drug Metabolism and Disposition, 2 (6), 583e588.

Ellman, G.L., Courtney, K.D., Andres, V. &Featherstone, R.M. (1961). A new and rapid colorimetric determination of acetylcholineserase activity. Biochemical Pharmacology, 7, 88e95. <https://doi.org/10.1016/0006-2952(61)90145-9>

Förlin, L., Andersson, T., Goksøyr, A., & Zhang, Y. (1988). Hepatic microsomal cytochromes P-450 from BNF-treated perch. Marine Environmental Research, 24(1-4), 112.

Lowry, O.H., Rosebrough, N.I., Farr, A.L. & Randall, R.J., 1951. Protein measurement with Folin phenol reagent. Journal of Biology and Chemistry, 193, 265e275.

Martínez-Gómez, C., Fernández, B., Benedicto, J., Valdés, J., Campillo, J. A., León, V.M. & Vethaak, A.D. (2012). Health status of red mullets from polluted areas of the Spanish Mediterranean coast, with special reference to Portmán (SE Spain). Marine environmental research, 77, 50-59. <https://doi.org/10.1016/j.marenvres.2012.02.002>

Martínez-Gómez, C., Fernández, B., Robinson, C. D., Campillo, J. A., León, V. M., Benedicto, J., ... & Vethaak, A. D. (2017). Assessing environmental quality status by integrating chemical and biological effect data: The Cartagena coastal zone as a case. Marine environmental research, 124, 106-117. <https://doi.org/10.1016/j.marenvres.2016.04.008>

1. **Tables**

Table S1. Detection limits for the chemical compounds analyzed in eels (*Anguilla anguilla*).

| Polyciclic aromatic hydrocarbons | µg/kg  (w.w.) |  | Organochlorine compounds | µg/kg (w.w.) |  | Organophosphorous | µg/kg  (w.w.) |
| --- | --- | --- | --- | --- | --- | --- | --- |
| Fluorene | 0.02 |  | α-β-γ-HCH | 0.05 |  | Chlorpyrifos | 0.37 |
| Phenanthrene | 0.02 |  | HCB | 0.02 |  | DCPA (Dacthal) | 0.06 |
| Anthracene | 0.04 |  | Aldrin | 0.1 |  | Fenchlorphos | 0.18 |
| Fluorantene | 0.04 |  | Isodrin | 0.1 |  | Pendimethalin | 0.54 |
| Pyrene | 0.04 |  | trans-NNC | 0.04 |  | Trichloronate | 0.18 |
| Benzo[a]anthracene | 0.05 |  | Dieldrin | 0.09 |  | Prothiofos | 0.85 |
| Chrysene | 0.1 |  | Endrin | 0.09 |  |  |  |
| Benzo[e]pyrene | 0.12 |  | *p,p'*- DDE | 0.03 |  |  |  |
| Benzo[b]fluoranthene | 0.03 |  | *p,p'* -DDD | 0.05 |  |  |  |
| Benzo[k]fluoranthene | 0.04 |  | *o,p'* -DDT | 0.07 |  |  |  |
| Benzo[a]pyrene | 0.04 |  | *p,p'* -DDT | 0.07 |  |  |  |
| Benzo[g,h,i]perylene | 0.05 |  | CB 28 | 0.04 |  |  |  |
| Dibenzo[a,h]anthracene | 0.05 |  | CB 52 | 0.04 |  |  |  |
| Indeno[1,2,3-c,d] pyrene | 0.09 |  | CB 101 | 0.04 |  |  |  |
|  |  |  | CB 105 | 0.03 |  |  |  |
|  |  |  | CB 118 | 0.03 |  |  |  |
|  |  |  | CB 138 | 0.04 |  |  |  |
|  |  |  | CB 153 | 0.03 |  |  |  |
|  |  |  | CB 156 | 0.03 |  |  |  |
|  |  |  | CB 180 | 0.03 |  |  |  |

Table S2. Detailed information of sampling sites and captures of European eel (*Anguilla anguilla*) from four sites in Mar Menor lagoon (SE Spain). SE= standard error of the mean; w.w.= wet weight); U= Undefined; F= female; M= male

| Mar Menor lagoon | Sampling  site | Sampling date | SW temperature | N | Age  (years) | Lentgh  (cm) | Eviscerated weight (g) | Sex  U / F / M | Yellow  stage | Silvering  stage | Silver  stage |
| --- | --- | --- | --- | --- | --- | --- | --- | --- | --- | --- | --- |
| Southern sub-area | S1 | 10/04/2014 | 16 ºC | 19 | 2 - 6 | 39.6 - 64.0 | 76.0 - 446.0 | 2 / 17 / 0 | 11 | 8 | 0 |
|  | S3 | 28/01/2015 | 13.8 ºC | 9 | 3 - 8 | 62.1-72.1 | 391.0 - 600.0 | 2 / 3 / 4 | 0 | 8 | 1 |
|  |  |  |  |  |  |  |  |  |  |  |  |
| Central sub-area | S2 | 25/02/2015 | 14.6 ºC | 15 | 3 - 6 | 45.7- 64.7 | 105.6 - 419.5 | 15 / 0 / 0 | 5 | 10 | 0 |
|  | S4 | 29/01/2015 | 14.2 ºC | 15 | 3 - 7 | 63.5- 80.5 | 391.0 - 888.0 | 5 / 5 / 5 | 0 | 6 | 9 |

Table S3. Concentration (mean ± SE) of polycyclic aromatic hydrocarbons (PAHs; µg·Kg-1 wet weight) in muscle and OH-PAH metabolites in bile (ng·µL^-1^ bile) of silvering European eels (*Anguilla anguilla*) captured in four different sites of the Mar Menor lagoon (SE Spain). SE= Standard Error of the mean. Superscript letters indicate significant differences between groups (1-way ANOVA; Kruskall wallis, T-test for the Mean; p-value ≤ 0.01)

|  | Southern basin | | Central basin | |
| --- | --- | --- | --- | --- |
|  | S1-Spring | S3-Winter | S2-Winter | S4 -Winter |
| PAHs | N=2 | N=8 | N=10 | N=6 |
| Fluorene | BDL | 0.76 ± 0.20 ^a^ | 0.87 ± 0.10 ^a^ | 0.57 ± 0.08 ^a^ |
| Phenanthrene | 0.08 ± 0.00 | 2.88 ± 0.24 ^a^ | 1.98± 0.26 ^a^ | 1.68 ± 0.28 ^a^ |
| Anthracene | BDL | 0.57 ± 0.14 ^a^ | 0.37 ± 0.05 ^a^ | BDL |
| Fluoranthene | 0.72 ± 0.08 | 1.29 ± 0.20 ^a^ | 0.51 ± 0.09 ^b^ | 0.37 ± 0.09 ^b^ |
| Pyrene | 0.79 ± 0.06 | 2.25 ± 0.42 ^a^ | 0.29 ± 0.05 ^b^ | 0.16 ± 0.05 ^b^ |
| Benzo(a)anthracene | BDL | BDL | 0.10 ± 0.02 ^a^ | 0.06 ± 0.03 ^a^ |
| Crysene | BDL | 0.20 ± 0.08 | BDL | BDL |
| Benzo(e)pyrene | BDL | 0.15 ± 0.14 ^a^ | 0.25 ± 0.13 ^a^ | BDL |
| Benzo(b)fluoranthene | BDL | 0.05 ± 0.05 ^a^ | 0.06 ± 0.01 ^a^ | 0.05 ± 0.02 |
| Benzo(k)fluoranthene | BDL | BDL | 0.04 ± 0.01 ^a^ | 0.06 ± 0.01 ^a^ |
| Benzo(a)pyrene | BDL | 0.05 ± 0.04 | BDL | BDL |
| Benzo(g,h,i)perylene | 0.15 ± 0.06 | BDL | BDL | BDL |
| Dibenzo(a,h)anthracene | 0.07 ± 0.04 | BDL | BDL | BDL |
| Indeno[1,2,3-c,d]pyrene | 7.79 ± 4.50 | 2.16 ± 1.88 ^a^ | BDL | 3.71 ± 3.71 ^a^ |
|  |  |  |  |  |
| OH-PAHs | N=8 | N=7 | N=9 | N=6 |
| 1-OHPhen | BDL | BDL | BDL | BDL |
| 1-OHPyr | 0.79 ± 0.11 ^a^ | 0.38 ± 0.04 ^a^ | 0.12 ± 0.01 ^b^ | 0.53 ± 0.09 ^a^ |

Table S4. Correlation between eviscerated weight, total length and muscle tissue concentration of contaminants of European eels (*Anguilla anguilla*) from Mar Menor lagoon (SE Spain). Not applicable= n.a.

|  | R-Pearson | Spearmen Rho | p-value | N |
| --- | --- | --- | --- | --- |
|  |  |  |  |  |
| Weight vs ∑9PCBs | -0.662 | n.a. | 0.011 | 20 |
| Weight vs pp-DDE | -0.574 | n.a. | 0.008 | 20 |
| Weight vs pp-DDD | n.a. | -0.550 | 0.012 | 20 |
| Weight vs Aldrin | -0.554 | n.a. | 0.011 | 20 |
|  |  |  |  |  |
| Length vs ∑9PCBs | -0.548 | n.a. | 0.012 | 20 |
| Length vs pp-DDE | -0.459 | n.a. | 0.042 | 20 |
| Length vs pp-DDD | n.a. | -0.595 | 0.006 | 20 |

Table S5. Concentrations (Mean ± SE and maximum value) of organochlorine compounds (ng·g^-1^ wet weight) and organophosphorus and other current-use pesticides (ng·g^-1^ wet weight) in silvering eels (*Anguilla anguilla*) from Mar Menor lagoon (SE Spain). ^(1)^ Sum of PCB28, PCB52, PCB101, PCB138, PCB153 and PCB180 according Commission regulation (EU) No 1259/2011 (maximum safe level ∑6CBs ≤ 300 ng·g^-1^ wet weight). ^(2)^ Sum of *op*-DDT, *pp*-DDT, *pp*-DDE and  *pp*-DDD according Commission regulation (86/363/EEC – in meat) (maximum safe level ∑DDTs ≤ 1000 ng·g^-1^ wet weight). SE= standard error of the mean; BDL= below detection limit; Superscript letters indicate significant differences between groups (1-way ANOVA; Kruskal Wallis, T-test for the Mean; p-value ≤ 0.01)

|  | Southern basin | | Central basin | |
| --- | --- | --- | --- | --- |
|  | S1-Spring | S3-Winter | S2-Winter | S4-winter |
| Sampling size | N=3 | N=3 | N=4 | N=4 |
| α-HCH | BDL | BDL | BDL | BDL |
| β-HCH | BDL | BDL | BDL | BDL |
| γ-HCH | BDL | BDL | BDL | BDL |
| HCB | 0.62 ± 0.13 ^a^ | 0.42 ± 0.12 ^a^ | 0.09 ± 0.08 ^a^ | 0.13 ± 0.05 ^a^ |
| Aldrin | 1.75 ± 0.51 ^a^ | 0.20 ± 0.09 ^a^ | 0.53 ± 0.38 ^a^ | 0.17 ± 0.06 ^a^ |
| Isodrin | BDL | BDL | BDL | BDL |
| Dieldrin | 3.64 ± 0.98 ^a^ | 1.19 ± 0.05 ^b^ | 1.09 ± 0.26^b^ | 1.50 ± 0.19 ^ab^ |
| Endrin | 0.68 ± 0.13 ^a^ | 0.71 ± 0.14 ^a^ | 0.81 ± 0.08 ^a^ | 0.83 ± 0.04 ^a^ |
| trans-nonachlor | 0.91 ± 0.13^a^ | 0.61 ± 0.04 ^a^ | 0.51 ± 0.08 ^a^ | 0.64 ± 0.03 ^a^ |
|  |  |  |  |  |
| CB28 | 0.28 ± 0.07 ^a^ | 0.11 ± 0.06 ^a^ | BDL | BDL |
| CB52 | 1.72 ± 0.51 ^a^ | 0.17 ± 0.08 ^a^ | 0.50 ± 0.041 ^a^ | 0.13 ± 0.05 ^a^ |
| CB101 | 15.11 ± 5.16 ^a^ | 0.95 ± 0.23 ^a^ | 8.59 ± 6.96 ^a^ | 1.33 ± 0.38 ^a^ |
| CB105 | 2.23 ± 0.80 ^a^ | 0.45 ± 0.08 ^a^ | 0.95 ± 0.41 ^a^ | 0.49 ± 0.12 ^a^ |
| CB118 | 15.23 ± 5.19 ^a^ | 1.64 ± 0.31 ^a^ | 7.84 ± 4.96 ^a^ | 1.55 ± 0.68 ^a^ |
| CB138 | 181.64 ± 60.32 ^a^ | 9.92 ± 4.01 ^ab^ | 100.93 ± 67.43 ^ab^ | 16.02 ± 3.97 ^ab^ |
| CB153 | 234.70 ± 78.87 ^a^ | 14.11 ± 5.51 ^a^ | 139.69 ± 92.75 ^a^ | 14.72 ± 8.25 ^a^ |
| CB 156 | 7.70 ± 2.65 ^a^ | 0.57 ± 0.13 ^a^ | 4.73 ± 3.54 ^a^ | 0.45 ± 0.11 ^a^ |
| CB 180 | 147.53 ± 49.57 ^a^ | 6.68 ± 2.55 ^ab^ | 88.91 ± 60.52 ^ab^ | 10.68 ± 2.84 ^ab^ |
| ∑ 9CBs | 606.14 ± 202.34 ^a^ | 34.59 ± 12.78 ^b^ | 352.14 ± 236.94 ^ab^ | 45.36 ± 14.98 ^ab^ |
| ∑ 6CBs ^(1)^ | 580.97 ± 194.11 ^a^ | 31.94 ± 12.36 ^b^ | 338.62 ± 228.04 ^ab^ | 42.87 ± 14.27 ^ab^ |
|  |  |  |  |  |
| pp-DDD | 17.08 ± 3.83 ^a^ | 2.36 ± 0.27 ^b^ | 4.89 ± 1.87 ^b^ | 3.40 ± 0.43 ^b^ |
| pp-DDE | 443.30 ± 151.93 ^a^ | 27.34 ± 6.03 ^b^ | 144.91 ± 63.63 ^ab^ | 56.78 ± 12.43 ^b^ |
| op-DDT | 1.60 ± 0.84 ^a^ | 0.60 ± 0.01 ^a^ | 0.88 ± 0.35 ^a^ | 0.47 ± 0.09 ^a^ |
| pp-DDT | 2.25 ± 0.73 ^a^ | 1.37 ± 0.29 ^a^ | 2.66 ± 1.08 ^a^ | 1.62 ± 0.15 ^a^ |
| ∑DDTs ^(2)^ | 464.22 ± 155.67 ^a^ | 31.67 ± 6.08 ^b^ | 153.33 ± 66.86 ^ab^ | 62.27 ± 12.89 ^b^ |
|  |  |  |  |  |
| Chlorpyrifos | 5.60 ± 0.61 ^a^ | 6.91 ± 2.52 ^a^ | 5.28 ± 2.97 ^a^ | 6.72 ± 4.51 ^a^ |
| Pendimethalin | BDL | 1.15 ± 0.32 ^a^ | BDL | 1.40 ± 0.07 ^a^ |

Table S6. Results of Correlation of organochlorine compounds with lipid content of European eels (*Anguilla anguilla*) from Mar Menor lagoon (SE Spain).

|  | | CB28 | CB52 | CB101 | CB118 | CB153 | CB105 | CB138 | CB156 | CB180 |
| --- | --- | --- | --- | --- | --- | --- | --- | --- | --- | --- |
| LIPID | Correlation coefficient | 0.085 | -0.252 | -0.349 | -0.260 | -0.253 | -0.083 | -0.290 | -0.266 | -0.254 |
|  | p-value | 0.722 | 0.283 | 0.131 | 0.268 | 0.283 | 0.728 | 0.214 | 0.257 | 0.280 |
|  | N | 20 | 20 | 20 | 20 | 20 | 20 | 20 | 20 | 20 |
|  |  |  |  |  |  |  |  |  |  |  |
|  |  | HCB | ALDRIN | t-NNC | ppDDE | DIELDRIN | ENDRIN | pp-DDD | op-DDT | pp-DDT |
|  | Correlation coefficient | 0.220 | -0.245 | **0.627**** | -0.292 | -0.075 | 0.327 | -0.417 | 0.035 | 0.214 |
|  | p-value | 0.352 | 0.297 | **0.003** | 0.212 | 0.753 | 0.160 | 0.068 | 0.885 | 0.364 |
|  | N | 20 | 20 | 20 | 20 | 20 | 20 | 20 | 20 | 20 |
|  |  |  |  |  |  |  |  |  |  |  |

Table S7. Concentrations of polychlorinated biphenyl compounds (PCBs; µg·Kg-1 lipid weight) in muscle of European eels (*Anguilla anguilla*) from Mar Menor lagoon (SE Spain). SE= Standard Error of the mean. EAC= Environmental Assessment Criteria for biota (OSPAR Commission, 2020) (1).

| PCB congener | EAC | Site | n | Mean | SE | Lower bound | Upper bound | Minimum | Maximum |
| --- | --- | --- | --- | --- | --- | --- | --- | --- | --- |
| CB28 | 67 | S1 | 5 | 1.34 | 0.13 | 0.97 | 1.71 | 0.88 | 1.67 |
|  |  | S2 | 5 | 0.00 | 0.00 | 0.00 | 0.00 | 0.00 | 0.00 |
|  |  | S3 | 5 | 0.34 | 0.21 | -0.24 | 0.91 | 0.00 | 0.85 |
|  |  | S4 | 5 | 0.00 | 0.00 | 0.00 | 0.00 | 0.00 | 0.00 |
| CB52 | 108 | S1 | 5 | 7.49 | 1.52 | 3.26 | 11.72 | 3.18 | 12.53 |
|  |  | S2 | 5 | 2.73 | 1.32 | -0.95 | 6.40 | 0.37 | 7.55 |
|  |  | S3 | 5 | 0.56 | 0.26 | -0.15 | 1.28 | 0.08 | 1.21 |
|  |  | S4 | 5 | 0.59 | 0.19 | 0.06 | 1.12 | 0.08 | 1.08 |
|  |  |  |  |  |  |  |  |  |  |
| CB101 | 121 | S1 | 5 | 62.09 | 15.60 | 18.78 | 105.41 | 22.17 | 113.91 |
|  |  | S2 | 5 | 43.81 | 22.42 | -18.44 | 106.06 | 6.87 | 128.60 |
|  |  | S3 | 5 | 3.95 | 0.72 | 1.93 | 5.96 | 2.23 | 6.37 |
|  |  | S4 | 5 | 4.45 | 0.81 | 2.21 | 6.69 | 2.97 | 7.14 |
| CB118 | 25 | S1 | 5 | 66.86 | 10.87 | 36.68 | 97.04 | 28.57 | 88.45 |
|  |  | S2 | 5 | 40.08 | 16.09 | -4.59 | 84.76 | 12.63 | 101.09 |
|  |  | S3 | 5 | 6.77 | 0.97 | 4.08 | 9.46 | 5.16 | 9.94 |
|  |  | S4 | 5 | 4.75 | 1.98 | -0.74 | 10.23 | 1.32 | 10.53 |
| CB138 | 317 | S1 | 5 | 816.91 | 170.11 | 344.62 | 1289.20 | 288.06 | 1297.63 |
|  |  | S2 | 5 | 487.25 | 222.27 | -129.87 | 1104.36 | 128.48 | 1338.79 |
|  |  | S3 | 5 | 39.25 | 11.97 | 6.02 | 72.47 | 19.42 | 85.09 |
|  |  | S4 | 5 | 66.33 | 12.13 | 32.65 | 100.01 | 28.47 | 93.83 |
| CB153 | 1585 | S1 | 5 | 1064.20 | 210.78 | 478.99 | 1649.41 | 382.90 | 1574.94 |
|  |  | S2 | 5 | 661.32 | 307.22 | -191.66 | 1514.30 | 180.96 | 1846.00 |
|  |  | S3 | 5 | 55.82 | 16.44 | 10.18 | 101.45 | 27.47 | 118.68 |
|  |  | S4 | 5 | 43.45 | 20.11 | -12.39 | 99.28 | 5.65 | 110.62 |
| CB180 | 469 | S1 | 5 | 685.49 | 149.15 | 271.37 | 1099.61 | 229.49 | 1050.58 |
|  |  | S2 | 5 | 400.22 | 202.82 | -162.90 | 963.35 | 103.81 | 1192.33 |
|  |  | S3 | 5 | 26.31 | 7.61 | 5.17 | 47.45 | 11.79 | 55.34 |
|  |  | S4 | 5 | 42.86 | 7.64 | 21.63 | 64.08 | 17.20 | 56.77 |

^(1)^OSPAR Commission, 2020. Publication Number 763/2020. 2019 Updated Audit trail of OSPAR EACs and other assessment criteria used to distinguish above and below thresholds. Substances and Eutrophication Series. 22pp. ISBN 978-1-913840-02-0.
